# Elucidating HLTF-Mediated DNA Fork Remodeling via Native Mass Spectrometry

**DOI:** 10.1021/jacs.5c22675

**Published:** 2026-04-24

**Authors:** Guan-Ting Lian, Hui Emmanuela Miriam, Yi-An Chen, Yen-Ju Chen, Peter Chi, Hsin-Yung Yen

**Affiliations:** 1 Institute of Biological Chemistry, 71561 Academia Sinica, Taipei 115201, Taiwan; 2 Institute of Biochemical Sciences, National Taiwan University, Taipei 106319, Taiwan; 3 Chemical Biology and Molecular Biophysics Program, Taiwan International Graduate Program, 71561 Academia Sinica, Taipei 115201, Taiwan; 4 Institute of Molecular and Cellular Biology, National Tsing Hua University, Hsinchu 300044, Taiwan

## Abstract

Replication fork reversal (RFR) is a crucial DNA damage tolerance mechanism that protects genome stability by remodeling DNA fork structures. The helicase-like transcription factor (HLTF) is one of the key components in the RFR process and is responsible for the conversion of a stalled replication fork into a four-way reversed fork, mediated by its ATPase activity. In contrast to a wealth of biochemical evidence depicting the biological activities of HLTF, very little information is available on how it molecularly and functionally interplays with DNA molecules. In this study, we employed native mass spectrometry (MS) to probe the stoichiometry of HLTF–DNA complexes and to elucidate their functional association with DNA fork remodeling. We revealed that HLTF exists as an inactive monomer with low accessibility to substrate ATP yet retains DNA fork binding activity. Intriguingly, in the presence of a DNA fork, monomeric HLTF forms a hetero protein–DNA complex that enhances its ATP accessibility, suggesting allosteric structural modulation through DNA fork interaction. Using both homologous and heterologous DNA forks, we further uncovered ATP-induced dimerization of HLTF and demonstrated its critical role in triggering the DNA unwinding activity of HLTF and subsequent DNA fork regression. Together, our findings provide unique insight into the molecular processes of a DNA remodeler and underscore the utility of native MS in probing the macro-assembly of protein–DNA complexes.

## Graphical abstract

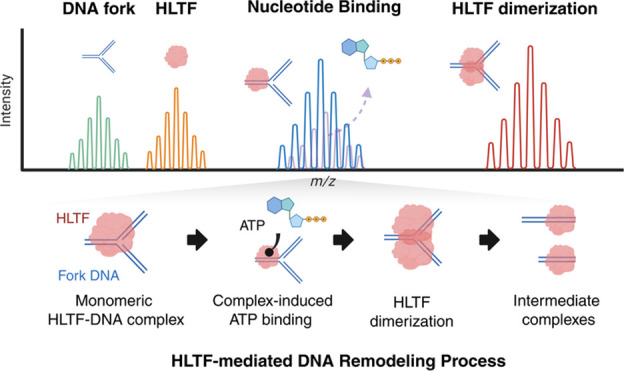

## Introduction

Replication fork reversal (RFR) is a key mechanism that responds to replication stress and safeguards genome stability. During RFR, crucial players are DNA fork remodelers from the sucrose nonfermenting family 2 (SNF2), which convert stalled three-way DNA junctions into four-way structures, stabilize replication forks, and prevent catastrophic outcomes such as double-strand breaks.^1^ The helicase-like transcription factor (HLTF), a member of the RAD5/16-like subgroup of SNF2 remodelers, catalyzes RFR through ATP hydrolysis.^2,3^ The human HLTF is composed of two conserved ATPase lobes, with a RING domain in between, for its ATPase activity. In addition, a HIP116, Rad5p N-terminal (HIRAN) domain is located at the N-termini of human HLTF with a function of substrate recognition by orienting the ATPase motor domain toward the stalled forks in order to initiate fork remodeling.^4,5^ Despite the biological importance of HLTF as a DNA fork remodeler, there are no high-resolution molecular structures of either full-length HLTF or HLTF–DNA complexes available, probably due to the dynamic nature of HLTF. Conventional biochemical assays, such as electrophoretic mobility shift assay (EMSA), have been instrumental in revealing domain-specific functions and fork regression activities across various DNA substrates.^4–9^ However, these methods could not resolve key aspects of HLTF–DNA interactions, such as complex heterogeneity, stoichiometry, and binding dynamics. Given the diverse nature of these interactions, a method that can offer detailed molecular insights is needed.

Native mass spectrometry (MS) has emerged as a powerful technique for the rapid, direct, and sensitive characterization of biomolecular interactions by preserving noncovalent assemblies under nondenaturing conditions.^10,11^ In comparison to established biochemical assays, most notably EMSA, native MS offers unique advantages in probing protein–DNA complexes, including complex heterogeneity,^12–16^ binding specificity,^13,15,17–21^ and stoichiometry.^13–24^ In this study, we extend the application of native MS to investigate the DNA remodeling process, focusing on the interaction between HLTF, a remodeler protein, and DNA fork substrates. A key technical challenge in applying native MS to protein–DNA complexes arises from the highly polyanionic nature of DNA, which favors ionization in negative ion mode electrospray ionization (ESI), whereas intact protein analysis is typically performed in positive ion mode ESI, creating an inherent incompatibility. A recent study of DNA assembly indicated the feasibility of using positive ion mode ESI for the characterization of DNA nanostructures;^25^ nevertheless, the positive polarity potentially has a stronger effect on the gas-phase structure of DNA complexes.^26^ Herein, we demonstrate the application of native MS with positive ion mode ESI to preserve DNA fork assembly and to elucidate protein-mediated DNA fork remodeling, exemplified by HLTF interactions with DNA fork substrates.

We generated a series of DNA substrates, including heterologous forks (HetF) and homologous forks (HomoF), which recapitulate cellular replication forks and enable monitoring of HLTF’s unwinding and fork reversal activities. In contrast to prior native MS studies that primarily examined simple single- or double-stranded oligonucleotides,^26^ our synthetic DNA fork structures mimic stalled replication forks, featuring a three-way junction containing either complementary or noncomplementary nascent strands. These structures effectively represent the paired parental strands and nascent strands at a stalled replication fork, providing a robust model for probing DNA–protein interactions and fork remodeling. Using native MS, we preserved the integrity of noncovalent DNA fork assembly and DNA-HLTF complexes in the gas phase, allowing accurate assessment of interaction dynamics and stoichiometry. Our findings indicate that DNA binding triggers conformational changes in HLTF, significantly altering its nucleotide affinity and promoting functional dimerization. These insights reveal key mechanisms of HLTF-mediated DNA remodeling and highlight the utility of native MS for studying complex DNA–protein interactions.

## Results and Discussion

### HLTF Exhibits Low Accessibility toward ATP

To investigate the molecular mechanism of HLTF-mediated fork remodeling, we first attempted to purify the full-length recombinant HLTF. The expression construct of full-length HLTF was introduced to Expi293F cells, and the protein was purified using tandem affinity chromatography followed by gel filtration (Figure S1a). The fork remodeling activity of purified HLTF was verified to ensure its functionality prior to MS characterization. Fork reversal assays were performed using HomoF82 substrates containing an 82-nt nascent strand (Tables S1 and S2). In these assays, HLTF catalyzed the unwinding of parental strands and the annealing of daughter strands, generating parental and nascent duplex DNA (Figure S1b) that could be resolved by polyacrylamide gel electrophoresis and visualized. As expected, HLTF promoted fork reversal in a concentration-dependent (Figure S1c) and ATP-dependent manner (Figure S1d).

To investigate the molecular mechanism of HLTF, we first characterized the purified recombinant HLTF using native MS. The mass spectrum of recombinant HLTF clearly indicates its monomeric state with a measured mass of 117,275.2 ± 1.1 Da (Figure 1a and Table S3). Intriguingly, a series of satellite signals was observed corresponding to mass shifts of approximately 78 Da, suggesting the potential of protein phosphorylation (Figure S1e). To examine this possibility, we incubated recombinant HLTF with λ-phosphatase (λ-PP), and the resulting mass spectra revealed a significant mass decrease following λ-PP treatment, confirming protein phosphorylation as reported previously (Figure S1e).^27,28^

**Figure 1:**
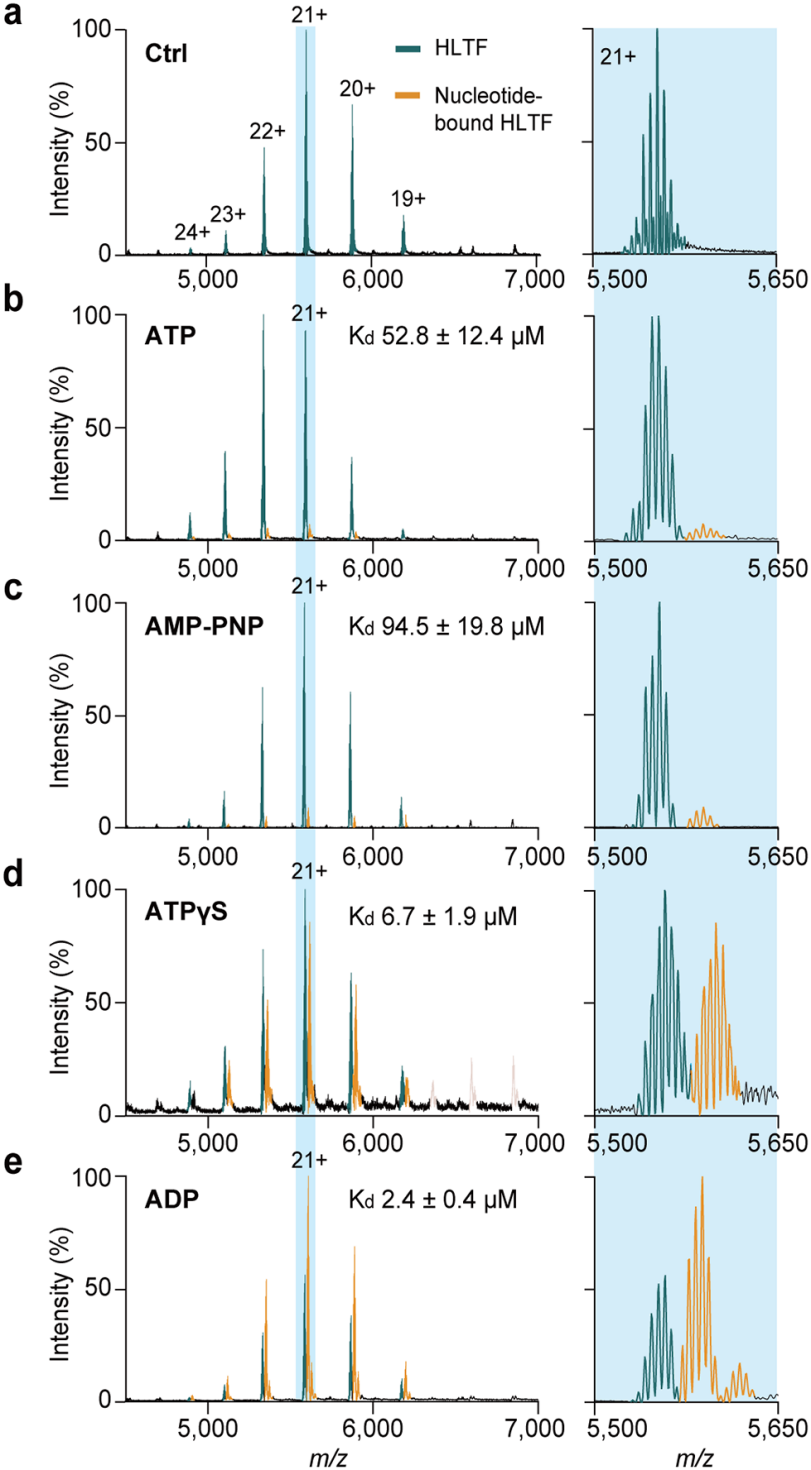
Native MS analysis of the nucleotide binding to monomeric HLTF. (a) Representative mass spectrum of purified full-length HLTF in ammonium acetate at 350 mM, pH 7.0. (b–e) Mass spectra of HLTF (8 μM) coincubated with ATP (b), AMP-PNP (c), ATPγS (d), or ADP (e), at equimolar concentrations relative to HLTF. Incubations were performed at room temperature for 5 min. The right panels show an expanded view of protein signals at the charge state 21+, indicating nucleotide binding and multiple phosphorylation on HLTF. The *K*_d_ values are estimated using a single-point approach, as described in the Experimental Section. All experiments were performed three times independently.

We next examined the nucleotide binding property of HLTF. Intriguingly, by comparing HLTF binding toward ATP and its analogs, we found that HLTF only moderately interacts with ATP, with a dissociation constant (*K*_d_) of 52.8 ± 12.4 μM using a single-point approach (Figure 1b).^29,30^ Based on the binding equilibrium at a single concentration point, the *K*_d_ for AMP-PNP was estimated at 94.5 ± 19.8 μM (Figure 1c). Interestingly, both ATPγS and ADP exhibit markedly higher affinity to HLTF, with *K*_d_ at 6.7 ± 1.9 and 2.4 ± 0.4 μM, respectively (Figures 1d,e). These affinity differences in nucleotide binding suggest that the geometry or electrostatic distribution of γ-phosphate could potentially be a determinant of HLTF’s nucleotide binding affinity. Overall, our results imply that HLTF structurally may reside in an inactive conformation with relatively low ATP accessibility, suggesting that additional structural mechanisms are required for HLTF activation.

### Optimization of Mass Spectrometry for DNA Fork Characterization

Conversion of a three-way homologous DNA fork into two annealed strands *in vitro* serves as a hallmark of HLTF-mediated fork reversal (Figure S1b).^1,4,6,31^ However, it remains unknown how HLTF exactly interacts with its DNA substrate to catalyze this process. We assembled a mimetic replication fork using four synthetic oligonucleotides in order to examine the interactions between HLTF and a DNA fork. Briefly, two overhang DNAs were initially created by annealing complementary 30-nucleotide daughter strands with 60-nucleotide parental strands. A fork junction was subsequently created by a second annealing event that brought two complementary overhang DNAs from the parental strands together. As a result, HomoF30 is a mimetic fork containing 30 bp duplex parental strand DNA with two 30 bp duplex arms representing the leading and lagging strands of the fork, which are complementary to each other, mimicking forks in the cell (Tables S1 and S2). Previous studies have demonstrated the applicability of native MS in characterizing higher-order DNA assemblies such as triplexes and G-quadruplexes; however, the progress on DNA fork analysis is limited.^25,26^

To optimize the analytical conditions for the DNA fork, we first investigated the effects of different volatile buffers. The buffer of HomoF30 was exchanged into ammonium bicarbonate (NH_4_HCO_3_), ammonium acetate (NH_4_OAc), and triethylamine acetate (TEAA) individually at a concentration of 350 mM for MS analysis (Figure 2a–d and Table S3). In positive ion mode, native MS revealed prominent signals corresponding to a double-stranded parental (d60), nascent (d30) DNA products, and overhang DNA (s60-s30) in ammonium bicarbonate, suggesting reduced stability of the fork structure in solution (Figure 2a). In contrast, the degree of fork disassembly was minimal under ammonium acetate or TEAA conditions, indicating their ability to preserve the integrity of the DNA fork both in solution and during gas-phase transition (Figure 2b,d).

**Figure 2:**
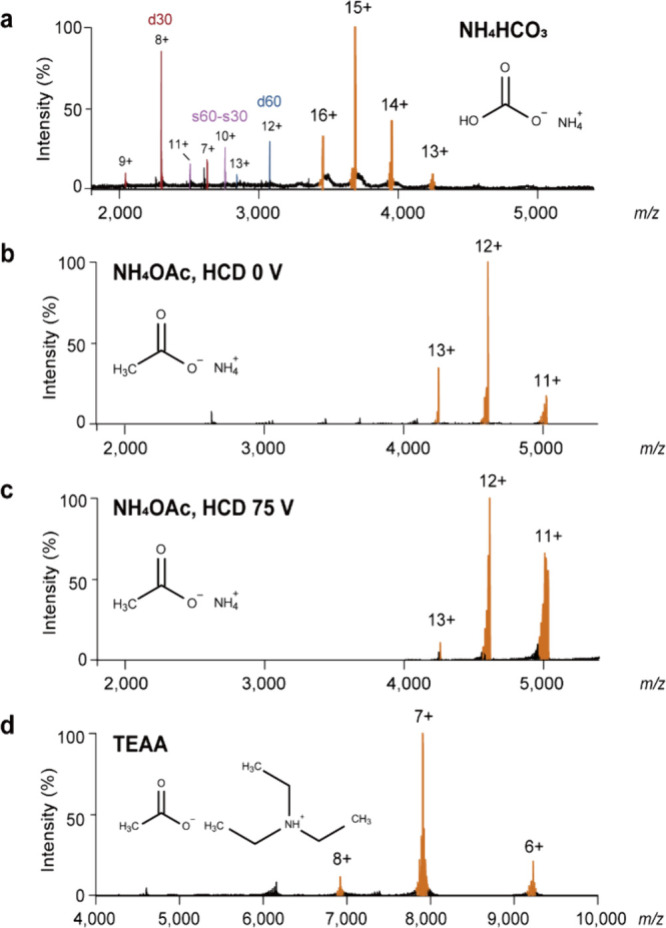
Characterization of homologous DNA fork under different buffer conditions. (a–d) Representative mass spectra acquired in positive ion mode ESI. DNA fork (10 μM) was analyzed in different volatile buffers, including ammonium bicarbonate (a), ammonium acetate (b), and triethylammonium acetate (d), each at 350 mM and pH 7. The dissociated products in (a) are annotated as d30, d60, and s60-s30. (c) Mass spectrum of the DNA fork in ammonium acetate under high collisional activation (75 V) in the HCD cell. All experiments were performed three times independently.

Intriguingly, under identical instrumentation settings, different buffer compositions produced distinct charge state distributions (CSDs). The charge envelope observed from ammonium acetate, ranging from 11^+^ to 13^+^, agreed well with the theoretical main charge state of 12.0^+^ predicated by the empirical equation for DNA (Figure 2b and Experimental Section), whereas the protein of equal mass exhibited a higher charge state of 14.1^+^.^26^ According to MD simulations and ion mobility spectrometry analysis, more compact DNA structures correspond to lower CSDs.^32^ The observation of a moderate charge increase, ranging from 13^+^ to 16^+^, for the DNA fork in ammonium bicarbonate suggests its extended or relaxed conformations (Figure 2a). In contrast, TEAA exhibited a significant charge-reducing effect, yielding charge states between 6^+^ and 8^+^, consistent with its previously reported property of reducing protein charges (Figure 2d).^33,34^ DNA molecules have been suggested to follow charging mechanisms during ESI similar to those of proteins, with their charge states decoupled in the solution phase. To test this notion, we performed the MS analysis at pH 4, and the CSD (11^+^ to 13^+^) of the DNA fork remained unchanged (Figure S2a). HomoF30 in ammonium acetate was also examined in negative ion mode ESI, resulting in a consistent CSD (11^–^ to 13^–^) compared with positive ion mode ESI (Figure S2b). Collectively, our results suggest a relation between CSDs and DNA fork structure, irrespective of ESI polarity and pH environment.

To further evaluate the gas-phase stability of the DNA fork, we introduced higher-energy collisional dissociation (HCD). By ramping up the HCD cell activation voltage, we found that the DNA fork assembly remained intact up to 75 V (Figure 2c). However, at 100 V, the spectral quality deteriorated significantly, suggesting partial disassembly or fragmentation of the DNA fork under high activation conditions (Figure S2c). Our findings support the applicability of positive ion mode ESI for probing HLTF–DNA fork interactions, and ammonium acetate was selected for subsequent native MS experiments to preserve DNA architecture and ensure compatibility with protein analysis.

### Interrogation of Interactions between HLTF and DNA Forks

While previous studies showed that the HIRAN domain of HLTF recognizes the 3′-hydroxyl end of single-stranded DNA and binds dsDNA,^4,5,8,9,35^ how full-length HLTF forms a complex with a DNA fork is less understood. To elucidate the mechanism underlying complex formation between HLTF and the DNA fork, we first investigated their interactions in the absence of ATP. Following a series of optimizations of experimental conditions, including the molar ratio of HLTF/DNA fork mixture, duration of reaction, concentration of volatile buffer, ESI polarity, and ion transmission, we successfully recorded the native MS spectrum of the HLTF–DNA fork complex (Figure 3a and Table S4). In addition to the predominant 1:1 HLTF–DNA fork complex, we detected a minor but well-resolved population corresponding to a 2:1 HLTF–DNA fork complex. The CSDs of the 1:1 and 2:1 complexes, respectively, ranged from 22^+^ to 27^+^ and 32^+^ to 34^+^, closely aligned with the estimated main charge states of the protein (25.3^+^ for the 1:1 complex and 33.2^+^ for the 2:1 complex), rather than those expected for DNA (21.7^+^ and 28.4^+^).^26^ Given that the CSD of protein–DNA complexes varies depending on their properties, we speculated that the exposed negative charges of the fork are effectively shielded upon HLTF binding, thereby mitigating the typical compaction effect observed for DNA in the positive ion mode ESI.

**Figure 3:**
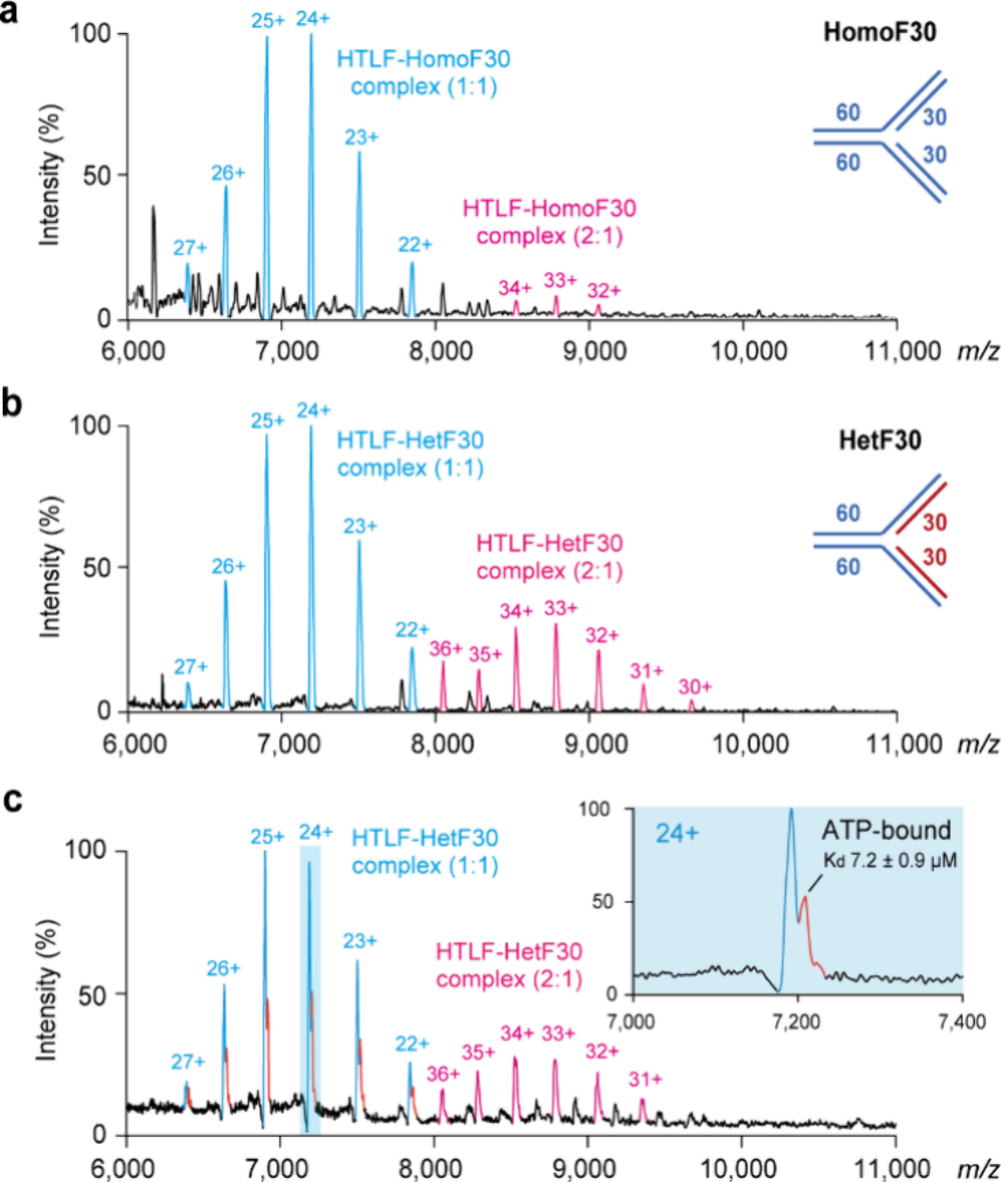
Native MS analysis of HLTF–DNA fork complexes in the absence of ATP. (a, b) Representative mass spectra showed the formation of HLTF complexes with HomoF30 (a) and HetF30 (b). The stoichiometry of observed species was assigned to 1:1 and 2:1 HLTF-DNA fork complexes. (c) Native MS analysis of the HLTF–HetF30 complex coincubated with ATP. HLTF (2.5 μM) and HetF30 (5 μM) were incubated at room temperature for 20 min, buffer-exchanged into ammonium acetate (350 mM, pH 7.0), and supplemented with ATP at equimolar concentrations to HLTF prior to MS analysis. The inner panel shows a zoomed-in view of the 24+ charge state, highlighting both apo and ATP-bound forms of the 1:1 HLTF–HetF30 complex. Each DNA fork binding assay was performed in three independent experiments.

The sequence homology of nascent strands of DNA fork has been reported as an essential component for HLTF-mediated fork reversal.^4,6^ To investigate its role in HLTF–DNA fork complex formation, we designed HetF30, a DNA fork with an architecture identical to HomoF30 but lacking sequence complementarity between the leading and lagging strands (Tables S1 and S2). Native MS analysis revealed that HLTF retains its activity in forming 1:1 complexes with HetF30, while more prominent 2:1 complexes were shown (Figure 3b). These results indicate that DNA sequence homology is not required for HLTF recognition of a stalled replication fork and that HetF30 recapitulates patterns comparable to those observed in HomoF30.

### Mass Spectrometry Revealed Greater ATP Accessibility in the HLTF–DNA Fork Complex

The ATP-independent complex formation observed between HLTF and DNA fork indicates that DNA binding itself is a crucial step in HLTF activation. Given the low ATP binding affinity of the HLTF monomer (Figure 1b), we next examined whether DNA binding modulates ATP accessibility of HLTF. This idea is consistent with the earlier finding that HLTF’s ATPase activity requires DNA substrates.^4^ The noncomplementary strand sequence of HetF30 is expected to prevent HLTF-mediated fork reversal induced by ATP, thereby enabling capture of ATP binding to the HLTF–DNA complex.

To probe ATP binding of the HLTF–DNA fork complex, recombinant HLTF was incubated with HetF30 under the same conditions described in Figure 3b, and ATP was supplied immediately prior to MS analysis in order to avoid its hydrolysis by HLTF. The mass spectrum revealed a distinct species corresponding to the ATP-bound HLTF–DNA fork complex. Quantitative analysis showed that the relative abundance of ATP-bound HLTF increased from 6.1% for HLTF alone (relative to its 100% apo population) to 46.6% when HLTF was bound to the DNA fork at an equimolar ATP ratio, indicating a substantial enhancement in ATP accessibility upon DNA fork engagement. The estimated *K*_d_ value of the ATP-bound HLTF–DNA fork complex is 7.2 ± 0.9 μM, indicating that ATP binds with much higher affinity to the HLTF–HetF30 complexes (Figure 3C). Although the structural details of full-length HLTF are yet unavailable, previous investigations on the motor domain of Snf2 from *Saccharomyces cerevisiae*, which shares a similar ATPase architecture, were interpreted as two conformations: the open state when binding to ADP and the closed state when binding to ATP analogs, ADP-BeFx.^36^ The binding of a DNA substrate shifts the structural equilibrium toward the closed configuration, thereby increasing ATPase activity. We speculate that HLTF may possess a regulatory mechanism similar to that of Snf2, controlling its conformation and activity by interacting with DNA fork.

### Stoichiometric Regulations of HLTF–DNA Complexes Induced by ATP

Biochemical studies have suggested a strong association between ATPase activity of HLTF and DNA fork remodeling.^5,6^ In light of the capability of capturing the HLTF–DNA fork complex by MS, we next investigated its stoichiometry regulations associated with ATP-induced fork reversal. The mixture of recombinant HLTF and HomoF30 was coincubated with ATP prior to MS analysis. The acquired mass spectrum resolved the multiple mass species corresponding to various protein–DNA complexes (Figure 4a). In addition to the 1:1 HLTF–HomoF30 complex as observed under ATP-free conditions, HLTF complexes with either a double-stranded parental (d60) or nascent DNA (d30) were detected in the presence of ATP, suggesting the formation of intermediate complexes during DNA fork remodeling (Figure 4a). The intermediate complexes observed align with the biochemical activity of HLTF, which remodels homologous DNA forks into double-strand products (Figure S1b–d).^6^ Intriguingly, a predominant mass species equivalent to a complex composed of two HLTF and one d30 was detected post ATP-incubation, indicating that the dimerization of HLTF plays a functional role in DNA fork remodeling (Figure 4a).

**Figure 4:**
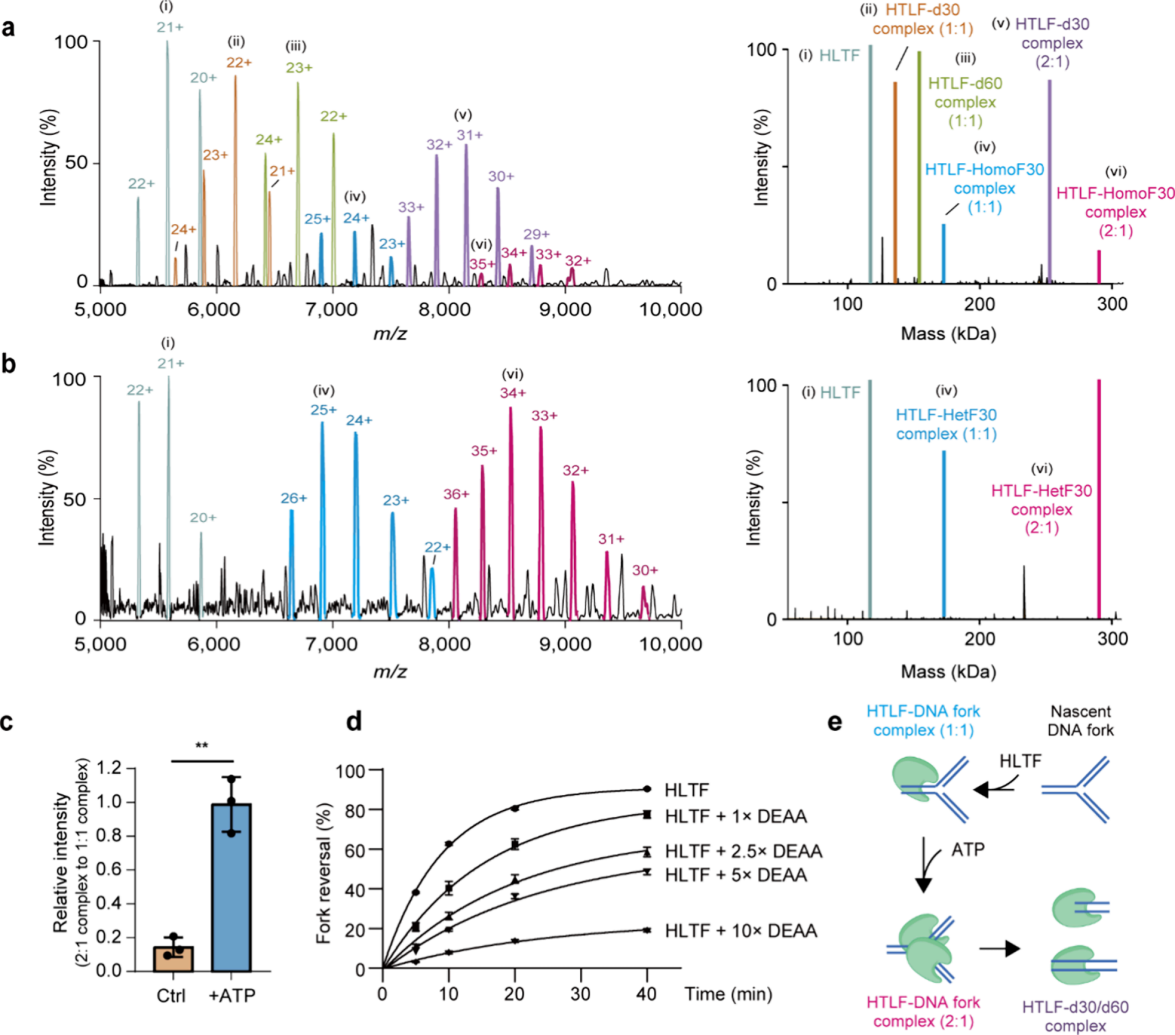
Native MS reveals ATP-induced stoichiometric modulations of HLTF–DNA fork complexes. (a) Incubation of HLTF and HomoF30 in the presence of ATP reveals multiple complexes corresponding to (i) monomeric HLTF, (ii) HLTF–d30 complex (1:1), (iii) HLTF–d60 complex (1:1), (iv) HLTF–HomoF30 complex (1:1), (v) HLTF–d30 complex (2:1), and (vi) HLTF–HomoF30 complex (2:1). The deconvoluted spectrum in the mass domain is shown in the right panel. (b) ATP induces dimerization of HLTF in complex with HetF30. The right panel presents the deconvoluted mass spectrum. (c) Quantification of HLTF–HetF30 complexes across different stoichiometries reveals increased HLTF dimerization following ATP treatment. Data are shown as mean ± SD from three replicates. (d) Competition with the HLTF-DEAA mutant in the fork reversal assay reduces wild-type HLTF activity, suggesting the importance of HLTF dimerization for fork reversal. Data are shown as mean ± SEM from three replicates. (e) Schematic illustration of ATP-dependent HLTF dimerization during fork remodeling. Unpaired *t* test, **P < 0.01.

To confirm the dimerization observed in HomoF30 after the addition of ATP, an analogous experiment was performed by incubating HLTF with HetF30 in the presence of ATP. We previously showed that HetF30 exhibits binding patterns similar to those of HomoF30 in the absence of ATP. Its heterologous sequence prevents HLTF from performing fork reversal and, therefore, is an ideal substrate for studying stoichiometric modulations of the HLTF–DNA fork complex prior to accomplishing DNA remodeling. The resulting mass spectrum revealed that the stoichiometry of HLTF–HetF30 complexes closely resembled those in the ATP-free condition, with no detectable remodeling products (Figure 4b). However, the intensity ratio between 2:1 and 1:1 complexes was significantly increased, indicating the increased HLTF dimerization upon ATP treatment (Figure 4c).

Considering the enhanced dimerization of HLTF upon ATP treatment with the HetF30 substrate and the presence of dissociated HomoF30 complexes bound to dimeric HLTF, we sought to test the functional link between HLTF dimerization and fork reversal activity. Here, we employed the ATPase-dead HLTF-DEAA mutant, which has been demonstrated to exhibit no fork reversal activity.^5,6^ We hypothesized that if dimerization is critical for HLTF’s fork reversal activity, and then competition between wild-type HLTF and the ATPase-dead DEAA mutant should markedly reduce activity, as fewer functional dimers would form. Consistent with this prediction, competition with a 2.5-fold excess of DEAA at the 40 min time point reduced activity by ∼32%, while a 10-fold excess nearly abolished HLTF’s fork reversal activity (Figure 4d). In contrast, RecG, the *Escherichia coli* fork reversal enzyme that functions as a monomer *in vitro* was only minimally affected even when competed with a 10-fold excess of its ATPase-dead mutant.^37^ Collectively, our native MS and biochemical analyses suggest that HLTF dimerization plays an important role in the DNA remodeling process and that its formation relies on the binding of ATP to HLTF (Figure 4e), consistent with previous studies of dimerization of other SWI2/SNF2 family proteins.^36,38^

### HLTF Inheres Unwinding Activity Induced by ATP

The initial step in fork reversal is believed to be the unwinding of nascent DNA strands from the parental strands. To test this hypothesis, an unwinding assay was performed with a DNA fork containing 15 nucleotides of heterologous nascent strands (HetF15). Our results showed that HLTF is capable of unwinding leading strand and lagging strand. As expected, ATP binding and hydrolysis are important for HLTF unwinding activity, as the absence of ATP or other nonhydrolyzable ATP analogs fails to show any unwinding activity from HLTF (Figure 5a). Note that the unwinding activity was not observed with the HetF30 substrate; we reasoned that HLTF may be only capable of unwinding shorter DNA strands.

**Figure 5:**
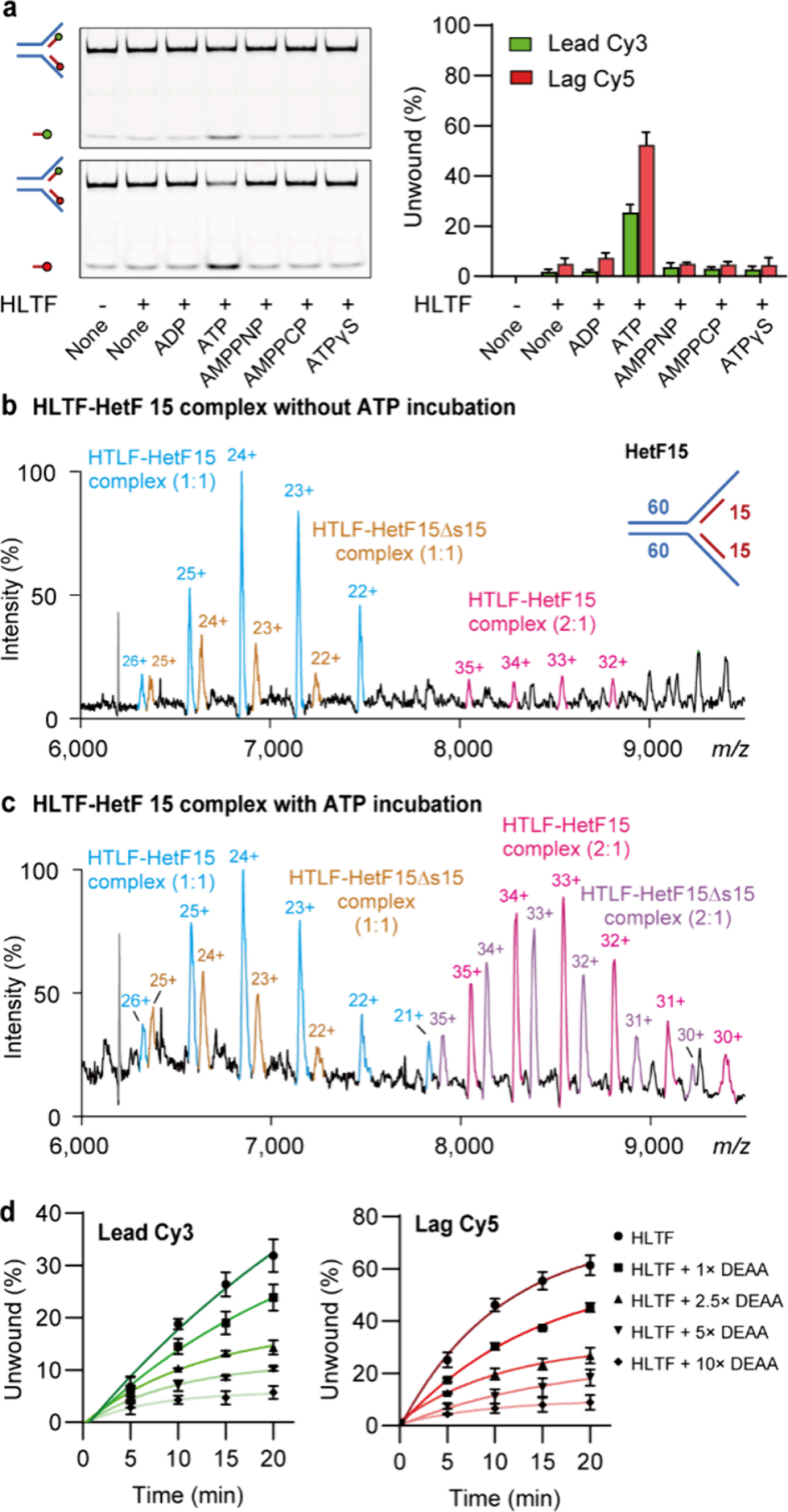
HLTF exhibits intrinsic unwinding activity on the DNA fork substrate HetF15. (a) DNA unwinding assay indicates that HLTF exhibits activity to unwind both leading and lagging strands of HetF15, only in the presence of ATP. (b, c) Representative mass spectra of 1:1 and 2:1 HLTF–HetF15 complexes in the absence of ATP (b) and with 10 μM ATP (c). Satellite signals adjacent to the 1:1 and 2:1 complexes correspond to HLTF bound to HetF15 lacking a nascent strand (HetF15Δs15). Reactions were carried out at room temperature for 20 min, and MS analysis was performed in ammonium acetate (350 mM, pH 7). The experiments were repeated independently three times. (d) Competition with the HLTF-DEAA mutant in the DNA unwinding assay reduces wild-type HLTF unwinding of both leading (left) and lagging (right) strands, suggesting the importance of HLTF dimerization for unwinding activity. Data are shown as mean ± SEM from three replicates.

To further investigate the stoichiometric relationship between HLTF-mediated unwinding activity and the short DNA fork substrates, we next attempted to probe the interactions between HLTF and HetF15 using mass spectrometry. First, HLTF was able to form complexes with HetF15 in the absence of ATP. The result indicated that, similar to HomoF30 and HetF30, HLTF retained its activity in forming 1:1 and marginal 2:1 complexes with HetF15 (Figure 5b). Second, when ATP was added, the mass spectrum revealed a similar phenomenon of increased HLTF dimerization (Figure 5c). Notably, additional mass species were detected, corresponding to HetF15 lacking a nascent strand (HetF15Δs15) in complex with the HLTF monomer and dimer. The intensity of HLTF–HetF15Δs15 complexes was profoundly enhanced by ATP, particularly for 2:1 HLTF–DNA fork complexes. The MS results are consistent with the unwinding activity of HLTF and suggest ATP and protein dimerization as critical factors in this process.

To further confirm that the dimerization of HLTF is important for HLTF unwinding activity, competition with HLTF-DEAA in unwinding assay was conducted. The result showed that both leading and lagging strand unwinding exhibited ∼50% reduced activity when competed with a 2.5-fold excess of DEAA at the 20 min time point (Figure 5d). Overall, these results suggest that HLTF exhibits intrinsic DNA unwinding activity capable of unwinding short DNA segments as an initial step in fork reversal. It is important to note that although monomeric HLTF binding to DNA fork can be detected, whether this monomeric fork is capable of unwinding duplex DNA and catalyzing fork reversal is still unclear and therefore would require further examination.

## Conclusions

Here, using native MS, we demonstrated that HLTF–DNA fork assembly can be preserved in the gas phase, enabling elucidation of the molecular mechanism underpinning HLTF-mediated DNA remodeling. MS characterization revealed that inactive HLTF is capable of binding to DNA forks and predominantly forming a 1:1 complex in nucleotide free conditions. Our results agree with previous EMSA analysis that full-length HLTF and DNA fork can form multiple protein–DNA complexes.^4,6^ Notably, we revealed that the DNA fork itself functions as an allosteric modulator for full activation of HLTF. Interactions of HLTF with DNA fork significantly increase its binding affinity toward ATP and possibly ATPase activity. Given that the ATPase activity is essential for HLTF-mediated fork remodeling, we conclude that RFR is tightly controlled by the presence of stalled forks in the nucleus. Moreover, MS characterization unexpectedly revealed the dimerization behavior of HLTF and its reliance on both DNA fork and ATP binding. The presence of the HLTF dimer in complex with two nascent strand products from a homologous DNA fork under ATP treatment, along with our biochemical assay using an ATPase-dead mutant as a competitor, further highlights the critical role of HLTF dimerization in DNA fork remodeling. Moreover, the observation of a low-populated 2:1 inactive HLTF–DNA fork complex suggests that HLTF recognition of the DNA fork may involve mechanisms beyond the established role of HIRAN in binding the free ends of nascent strands. Further investigations are necessary to define the structural details of dimeric HLTF and DNA fork, and to elucidate the molecular processes underlying DNA fork remodeling.

In conclusion, we proposed that HLTF undergoes conformational changes upon binding to DNA forks, which enhances its ATP binding and protein dimerization to facilitate efficient fork regression, including DNA unwinding activity. Our work presented in this study highlights native MS as a powerful tool for probing DNA–protein interactions and their functional readouts. Given that the replication stress response is highly coordinated with the interactions among DNA and various protein components such as RAD51 and BRCA1/2,^1^ our study paves the way for future research to delineate the molecular details of these complex events. Moreover, the findings here advance our understanding of genome stability mechanisms and potentially provide a foundation for exploring therapeutic strategies targeting DNA repair pathways in cancer and other diseases.

## Experimental Section

### Expression and Purification of Wild-Type Human HLTF and ATPase-Dead Mutant

Expi293F cells (Thermo Fisher) were transfected with pcDNA-(His)_6_-Flag-HLTF or pcDNA-(His)_6_-Flag-HLTF (DEAA) expression plasmids according to the instruction manual from the ExpiFectamine 293 kit (Thermo Fisher). The cells were harvested 48 h after transfection by centrifugation. Protein purification was performed at 4 °C. Cells were lysed using buffer A (25 mM Tris–HCl, pH 7.5, 10% glycerol, 500 mM KCl, 0.01% Igepal-CA-630, 0.5 mM EDTA, 10 mM ATP, and 10 mM MgCl_2_) supplemented with 1 mM β-mercaptoethanol and protease inhibitors (2 mM PMSF, 2 mM benzamidine and 1 μg/mL each of aprotinin, chymostatin, leupeptin, and pepstatin A) and sonicated. Samples were centrifuged at 100,000*g* for 1 h to separate pellet and supernatant. The supernatant was mixed with Ni^2+^-NTA agarose resin (QIAGEN) and incubated for 3 h at 4 °C. The resin was washed with buffer A supplemented with 1 mM β-mercaptoethanol and 10 mM imidazole and with buffer B (25 mM Tris–HCl, pH 7.5, 10% glycerol, 300 mM KCl, 0.01% Igepal-CA-630, 0.5 mM EDTA, 10 mM ATP, and 10 mM MgCl_2_) supplemented with 1 mM β-mercaptoethanol and 20 mM imidazole. Protein was eluted with buffer B supplemented with 200 mM imidazole. The eluted protein was bound with antiflag M2 affinity gel (Sigma-Aldrich) overnight. Protein-bound resin was washed with buffer B. Proteins were eluted with buffer B supplemented with 3× Flag peptide (100 μg/mL). Eluted protein is then supplemented with 1 mM β-mercaptoethanol. Proteins were separated further by gel filtration Superose 6 Increase 10/300 GL (GE Healthcare) using buffer C (25 mM Tris–HCl, pH 7.5, 10% glycerol, 300 mM KCl, 0.01% Igepal-CA-630, 1 mM β-mercaptoethanol, and 0.5 mM EDTA). The fractions containing the purified protein were collected, concentrated, and stored at −80 °C. The same purification protocol was applied to the DEAA mutant variant. For *in vitro* dephosphorylation, lambda protein phosphatase (λ-PP, New England Biolabs) was used to remove phosphorylation. Purified protein (10 μM) was incubated with 200 units of λ-PP lambda protein phosphatase and 1× MnCl_2_ (provided in the reaction kit) in buffer C. The reaction was performed at room temperature for 30 min.

### DNA Fork Substrate Preparation

The DNA oligonucleotides and the fluorescently labeled DNA oligonucleotides were purchased from Genomics. The sequences of synthetic DNA are provided in Table S1.

HomoF30 is annealed by incubating RF1 + RF1C.30 and RF2 + RF2C.30 separately in annealing buffer (50 mM Tris–HCl, pH 7.5, 10 mM MgCl_2_, 100 mM NaCl, and 1 mM DTT) and heated at 80 °C for 3 min and then transferred to 65 °C for 30 min and cooled down to room temperature overnight. The two overhang DNAs are then mixed and incubated at 37 °C for 30 min before the sample was run on 10% TBE (90 mM Tris–boric acid, pH 8.0, and 2 mM EDTA) polyacrylamide gel, and the area corresponding to the correct size of the product was excised and subsequently filter-dialyzed into TE buffer (10 mM Tris–HCl pH 8.0, and 0.5 mM EDTA) at 4 °C using an Amicon ultra-4 concentrator (Millipore, NMWL 10 kDa). HomoF82 is annealed by incubating Cy3-RF4 + RF4C.82 and RF5 + Cy3-RF5C.82 separately in annealing buffer. The rest of the steps followed HomoF30 substrate preparation.

HetF30 and HetF15 were annealed by incubating all four strands of synthetic DNA, which are RF1 + RF1C.30 + RF3 + RF3C.30 and RF1 + RF1C.15 + RF3 + RF3C.15, respectively, in annealing buffer and heated at 80 °C for 3 min, and then transferred to 65 °C for 30 min and cooled down to room temperature overnight. The sample was resolved on a 10% TBE–PAGE gel and purified using the same procedure as for HomoF30. Fluorescent-labeled HetF15 is annealed in the same way as HetF15 but by incubating with fluorescent-labeled nascent strands: RF1 + RF1C.15-Cy3 + RF3 + RF3C.15-Cy5.

### Fork Reversal Assay

Fluorescently labeled DNA fork substrate HomoF82 (15 nM) was incubated with HLTF in buffer D (35 mM Tris–HCl, pH 7.5, and 1 mM DTT) supplemented with 100 mM KCl, 0.1 μg/μL BSA, 2 mM MgCl_2_, and 2 mM nucleotides (ATP or AMP-PNP or ADP or ATPγS as indicated) at 37 °C for 40 min. The reaction was mixed with a 2.5 μL termination buffer (48 mM EDTA, 0.02% SDS, and 0.64 mg/mL proteinase K) and incubated at 37 °C for 15 min to stop the reaction. Three μL of loading dye is added, and the reaction mixtures are resolved in a 6% TBE–PAGE gel with 1× TBE buffer at 110 V for 60 min on ice. Gels were analyzed using an Amersham Typhoon Biomolecular Imager with a Cy3 570BP20 560–580 nm filter. Amersham ImageQuant software was then used to quantify the signal intensity of DNA species.

For the fork reversal competition assay, HLTF and the HLTF-DEAA mutant were preincubated on ice for 5 min in buffer D supplemented with 100 mM KCl, 0.1 μg/μL BSA, 2 mM MgCl_2_, and 2 mM ATP. The fluorescently labeled fork substrate HomoF82 (15 nM) was then added. At each indicated time point, 10 μL samples were collected, and reaction termination and subsequent steps were performed as described for the fork reversal assay above. A nonlinear one-phase association model in GraphPad Prism 10.6.1 was used to fit the smooth curve.

### DNA Unwinding Assay

Fluorescently labeled DNA fork substrate HetF15 (10 nM) was incubated with HLTF in buffer D supplemented with 50 mM KCl, 0.1 μg/μL BSA, 2 mM MgCl_2_, 2 mM ATP, and 100 nM trap DNA (RF1C.15+RF3C.15) at 37 °C for 20 min. The reaction was mixed with a 2.5 μL termination buffer and incubated at 37 °C for 15 min to stop the reaction. 3 μL of loading dye is added, and the reaction mixtures are resolved in a 10% TBE–PAGE gel with 1× TBE buffer at 110 V for 60 min on ice. Gels were analyzed using an Amersham Typhoon Biomolecular Imager with a Cy3 570BP20 560–580 nm and Cy5 670BP30; 655–685 nm filters. Amersham ImageQuant software was then used to quantify the signal intensity of DNA species. Unwinding activity was quantified by subtracting the signal of the no-protein control from that of the experimental samples.

For the DNA unwinding competition assay, HLTF and the HLTF-DEAA mutant were preincubated on ice for 5 min in buffer D supplemented with 50 mM KCl, 0.1 μg/μL BSA, 2 mM MgCl_2_, and 2 mM ATP. The fluorescently labeled fork substrate HetF15 (10 nM) along with 100 nM trap DNA (RF1C.15+RF3C.15) were then added. At each indicated time point, 10 μL samples were collected, and reaction termination and subsequent steps were performed as described for the DNA unwinding assay above. A nonlinear one-phase association model in GraphPad Prism 10.6.1 was used to fit the smooth curve.

### HLTF–DNA Fork Complex Formation

HLTF–DNA fork complexes were formed by incubating 2.5 μM purified HLTF with 5 μM DNA fork in buffer D supplemented with 0.1 μg/μL BSA and 2.5 mM MgCl_2_ at room temperature for 20 min. To capture intermediates or final products of DNA fork remodeling, 10 μM ATP was included in the incubation buffer under the same conditions. Following incubation, samples were buffer-exchanged into a volatile buffer (350 mM ammonium acetate, or as otherwise specified) using Zeba spin desalting columns (40 K MWCO; Thermo Fisher Scientific) according to the manual instructions prior to native MS analysis. To test the ATP binding affinity to the HLTF–DNA fork complex, 2.5 μM ATP and MgCl_2_ were added to the sample after buffer exchange, and the sample was immediately analyzed by native MS.

### Native Mass Spectrometry Analysis

DNA fork binding to HLTF was analyzed using a modified Q-Exactive mass spectrometer (UHMR, Thermo Fisher Scientific) after incubating purified HLTF with synthetic DNA fork in a 1:1 to 1:3 molar ratio at 4 °C in the protein buffer (35 mM Tris–HCl, pH 7.5, 100 mM KCl, and 2 mM MgCl_2_) for at least 20 min. Protein samples were buffer-exchanged into 350 mM ammonium acetate buffer, pH 7.5, loaded onto in-house-fabricated gold-coated emitters, and electrosprayed into UHMR. The MS parameters included spray voltage, 1.2–1.4 kV; capillary temperature, 200 °C; S-lens RF level, 200; resolution, 6250–12,500. To optimize high *m*/*z* range ion transmission within the mass spectrometer, a gentle voltage gradient (injection flatapole, inter flatapole, bent flatapole, and transfer multipole: 5.0, 4.0, 2.0, and 0 V, respectively) was applied. To facilitate desolvation and dissociate adducts, in-source CID 0 to 50 V, in-source trapping −50 to −150 V, and HCD collision energy 0 to 50 V were applied. The trapping gas pressure was set to 5.0. RAW files were analyzed and processed manually using Thermo Xcalibur Qual Browser (version 2.0.3) in conjunction with UniDec (Universal Deconvolution) software (version 8.0.3) for intensity quantification as needed. Measurement errors indicate differences in the peak centroids of the same mass species across various charged states.

The main charge states for complexes are respectively estimated based on the following equations: eq 1 (protein) and eq 2 (DNA):^26^

$$Z_{p}=0.048\times m^{0.52}$$ 1

$$Z_{d}=0.041\times m^{0.52}$$ 2

### Single-Point Approach for *K*_d_ Value Calculation

To assess the binding affinities of various nucleotides, purified HLTF or preincubated HLTF–DNA complexes were buffer-exchanged into a volatile buffer (350 mM ammonium acetate) using Zeba spin desalting columns (40 kDa MWCO; Thermo Fisher Scientific), and protein concentration was determined by UV absorbance at 280 nm. Following buffer exchange, nucleotides were added at the indicated concentrations. Mass spectra were acquired, and the relative abundances of the ligand-bound species Ab­(PL) and unbound species Ab­(P) were quantified using UniDec. The observed bound ratio *R* was calculated as in eq 3:

$$R=\frac{Ab\left(PL\right)}{Ab\left(P\right)}=\frac{\left[PL\right]}{\left[P\right]}$$ 3

The equilibrium dissociation constant *K*_d_ was then calculated using the following rearranged mass balance expression based on the total protein concentration [*P*]_0_ and ligand concentration [*L*]_0_ using eq 4:

$$K_{d}=\frac{{\left[L\right]}_{0}}{R}-\frac{{\left[P\right]}_{0}}{1+R}$$ 4

## Supplementary Material



## Notes

DNA fork designs (Tables S1 and S2) and the deconvoluted masses of HLTF–DNA fork assemblies (Tables S3–S6); additional experimental details for protein purification, characterization (Figure S1); and DNA fork measurement (Figure S2) (PDF)
