# Supplementary material for: Elucidating HLTF-Mediated DNA Fork Remodeling via Native Mass Spectrometry
Source: J Am Chem Soc. 2026 Apr 24;148(17):17707–16. doi: 10.1021/jacs.5c22675 (PMC13154178; doi:10.1021/jacs.5c22675)
Supplement: Supplementary file 1 [file ja5c22675_si_001.pdf]

Supporting Information for

Elucidating HLTF-mediated DNA Fork Remodeling via Native Mass Spectrometry

Guan-Ting Lian[1, 2, 3 §], Hui Emmanuela Miriam[1, 2, 3, 4 §], Yi-An Chen[1], Yen-Ju Chen[2], Peter Chi[1, 2, *], and Hsin-Yung Yen[1, 2, *]

[1] Institute of Biological Chemistry, Academia Sinica, Taipei 115201, Taiwan.

[2] Institute of Biochemical Sciences, National Taiwan University, Taipei 106319, Taiwan.

[3] Chemical Biology and Molecular Biophysics Program, Taiwan International Graduate Program, Academia Sinica, Taipei 115201, Taiwan.

[4] Institute of molecular and cellular biology, National Tsing Hua University, Hsinchu 30013, Taiwan

[§]G.-T.L. and H.E.M. contributed equally to this work.

*e-mail: hsinyungyen@gate.sinica.edu.tw; peterhchi@ntu.edu.tw

Table of Contents

Table S1. DNA strands used for fork assembly.

Table S2. Designs of DNA forks applied in this study.

Table S3. Molecular masses of HLTF and DNA forks measured by native MS.

Table S4. Molecular masses of HLTF-HomoF30 complex measured by native MS.

Table S5. Molecular masses of HLTF-HetF30 complex measured by native MS.

Table S6. Molecular masses of HLTF-HetF15 complex measured by native MS.

Figure S1. Characterization of purified recombinant HLTF.

Figure S2. Characterization of DNA fork substrates.

Table S1. DNA strands used for fork assembly.

Name	Length (nucleotides)	DNA sequences (5’ to 3’)	Fluorescence
RF1	60	ACGCTGCCGAATTCTACCAGTGCCTTGCTAGGACATCTT TGCCCACCTGCAGGTTCACCC	
RF1C.30	30	GGGTGAACCTGCAGGTGGGCAAAGATGTCC	
RF1C.15<br>RF1C.15-Cy3	15	TGGGCAAAGATGTCC	Cy3
RF2	60	GGGGTGAACCTGCAGGTGGGCAAAGATGTCCTAGCAAG GCACTGGTAGAATTCGGCAGCGT	
RF2C.30	30	GGACATCTTTGCCCACCTGCAGGTTCACCC	
RF3	60	CGATAGTCGGATCCTCTAGACAGCTCCATGTAGCAAGG CACTGGTAGAATTCGGCAGCGT	
RF3C.30	30	CATGGAGCTGTCTAGAGGATCCGACTATCG	
RF3C.15<br>RF3C.15-Cy5	15	CATGGAGCTGTCTAG	Cy5
Cy3-RF4	122	Cy3- CGTGACTTGATGTTAACCCTAACCCTAAGATATCGCGTT ATCAGAGTGTGAGGATACATGTAGGCAATTGCCACGTGT CTATCAGCTGAAGTTGTTCGCGACGTGCGATCGTCGCT GCGACG	Cy3
RF4C.82	82	CGTCGCAGCGACGATCGCACGTCGCGAACAACTTCAGC TGATAGACACGTGGCAATTGCCTACATGTATCCTCACAC TCTGA	
RF5	122	CGTCGCAGCGACGATCGCACGTCGCGAACAACTTCAGC TGATAGACACGTGGCAATTGCCTACATGTATCCTCACAC TCTGAATACGCGATATCTTAGGGTTAGGGTTAACATCAA GTCACG	
Cy3-RF5C.82	82	TCAGAGTGTGAGGATACATGTAGGCAATTGCCACGTGT CTATCAGCTGAAGTTGTTCGCGACGTGCGATCGTCGCT GCGACG	Cy3

Table S2. Designs of DNA forks applied in this study.

Name	Representative Drawing	Corresponding assay	Combined DNA
HomoF30	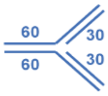	Fork reversal	(RF1+RF1C.30) + (RF2+RF2C.30)
HetF30	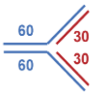	DNA binding analysis	RF1+RF1C.30 + RF3+RF3C.30
HetF15	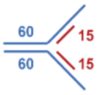	DNA unwinding (native mass)	RF1+RF1C.15 + RF3+RF3C.15
HetF15-Cy3 + Cy5	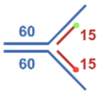	DNA unwinding (biochemical)	RF1+RF1C.15-Cy3 + RF3+RF3C.15-Cy5
HomoF82	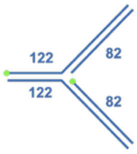	Fork reversal	(Cy3-RF4+RF4C.82) + (RF5+Cy3-RF5C.82)

Table S3. Molecular masses of HLTF and DNA forks measured by native MS.

	m/z value	Observed mass (Da)	Theoretical mass (Da)	ΔMass (Da), %
**HLTF**	4887.4 (24+)<br>5099.9 (23+)<br>5331.7 (22+)<br>5585.6 (21+)<br>5864.8 (20+)<br>6173.4 (19+)	117275.2 ± 1.1	117,261<br>(+5 phosphorylation)	13.6, 0.0%
**HetF30**	4260.0 (13+)<br>4615.0 (12+)<br>5034.4 (11+)	55367.2 ± 0.4	55,368	0.8, 0.0%
**HomoF30**	4260.1 (13+)<br>4615.1 (12+)<br>5034.5 (11+)	55368.5 ± 0.1	55,367	1.5, 0.0%
**d60**	2843.6 (13+)<br>3080.5 (12+)	36953.6 ± 0.34	36,954	0.4, 0.0%
**d30**	2047.3 (9+)<br>2302.9 (8+)<br>2631.8 (7+)	18415.5 ± 0.91	18,416	0.5, 0.0%
**s60-s30**<br>(RF1-RF1C.30)	2509.7 (11+)<br>2760.6 (10+)	27595.6 ± 0.38	27,596	0.4, 0.0%
**HetF15**	-	-	47,205	-
**HetF15Δs15**	-	-	41,908 (ΔRF1C15)<br>42,081 (ΔRF3C15)	-

Table S4. Molecular masses of HLTF-HomoF30 complex measured by native MS.

	m/z value	Observed mass (Da)	Theoretical mass (Da)	ΔMass (Da), %
**HLTF-HomoF30 complex (1:1)**	6640.6 (26+)<br>6907.2 (25+)<br>7194.2 (24+)<br>7507.6 (23+)<br>7847.2 (22+)	172637.3 ± 16.0	172,628<br>(+5 phosphorylation)	9.3, 0%
**HLTF-d60 complex (1:1)**	6169.9 (25+)<br>6427.7 (24+)<br>6707.0 (23+)<br>7014.2 (22+)<br>7346.1 (21+)<br>7714.3 (20+)	154250.2 ± 23.9	154,215<br>(+5 phosphorylation)	35.2, 0.0%
**HLTF-d30 complex (1:1)**	5653.4 (24+)<br>5898.2 (23+)<br>6169.9 (22+)<br>6462.2 (21+)<br>6784.3 (20+)	135671.7 ± 30.0	135,677<br>(+5 phosphorylation)	5.3, 0.0%
**HLTF-HomoF30 complex (2:1)**	8528.4 (34+)<br>8789.3 (33+)<br>9063.7 (32+)	289983.5 ± 46.4	289,889<br>(+5 phosphorylation)	84, 0.0%
**HLTF-d30 complex (2:1)**	7658.0 (33+)<br>7899.0 (32+)<br>8153.6 (31+)<br>8426.0 (30+)<br>8717.2 (29+)	252733.0 ± 32.1	252,938<br>(+10 phosphorylation)	205, 0.1%

Table S5. Molecular masses of HLTF-HetF30 complex measured by native MS.

	m/z value	Observed mass (Da)	Theoretical mass (Da)	ΔMass (Da), %
**HLTF-HetF30 complex (1:1)**	6394.5 (27+)<br>6642.1 (26+)<br>6907.8 (25+)<br>7195.5 (24+)<br>7508.0 (23+)<br>7850.1 (22+)	172661.6 ± 19.4	172,629<br>(+5 phosphorylation)	32.6, 0.3%
**HLTF-HetF30 complex (2:1)**	8057.7 (36+)<br>8289.1 (35+)<br>8531.8 (34+)<br>8791.2 (33+)<br>9066.9 (32+)<br>9359.7 (31+)<br>9670.9 (30+)	290081.5 ± 29.4	289,890<br>(+10 phosphorylation)	190.5, 0.1%

Table S6. Molecular masses of HLTF-HetF15 complex measured by native MS.

	m/z value	Observed mass (Da)	Theoretical mass (Da)	ΔMass (Da), %
**HLTF-HetF15 complex (1:1)**	6323.9 (26+)<br>6576.0 (25+)<br>6850.4 (24+)<br>7148.1 (23+)<br>7473.3 (22+)<br>7830.0 (21+)	164389.5 ± 12.1	164,466<br>(+ 5 phosphorylation)	76.7, 0.1%
**HLTF-HetF15Δs15 complex (1:1)**	6641.7 (25+)<br>6929.9 (24+)<br>7246.1 (23+)<br>7590.5 (22+)	159377.9 ± 12.0	159,169 (ΔRF1C15)<br>159,342 (ΔRF3C15)<br>(+ 5 phosphorylation)	208.9, 0.1%<br>35.9, 0.0%
**HLTF-HetF15 complex (2:1)**	8053.9 (35+)<br>8291.9 (34+)<br>8539.9 (33+)<br>8808.1 (32+)<br>9089.7 (31+)<br>9394.8 (30+)	281819.1 ± 49.3	281,727<br>(+ 10 phosphorylation)	92.1, 0.0%
**HLTF-HetF15Δs15 complex (2:1)**	7903.6 (35+)<br>8135.1 (34+)<br>8384.5 (33+)<br>8645.4 (32+)<br>8921.9 (31+)<br>9219.7 (30+)	276588.4 ± 41.9	276,430 (ΔRF1C15)<br>276,604 (ΔRF3C15)<br>(+ 10 phosphorylation)	158.4, 0.1%<br>15.6, 0.0%
**HLTF-HetF15 complex (2:2)**	8895.4 (37+)<br>9146.4 (36+)<br>9400.1 (35+)<br>9679.3 (34+)<br>9974.6 (33+)<br>10284.1 (32+)	329090.6 ± 88. 6	328,932<br>(+ 10 phosphorylation)	158.6, 0.1%
**HLTF-HetF15Δs15 complex (2:2)**	9001.6 (36+)<br>9257.9 (35+)<br>9534.7 (34+)<br>9821.8 (33+)<br>10129.3 (32+)	324070.5 ± 62.3	323,635 (ΔRF1C15)<br>323,808 (ΔRF3C15)<br>(+ 10 phosphorylation)	435.5, 0.1%<br>262.5, 0.1%

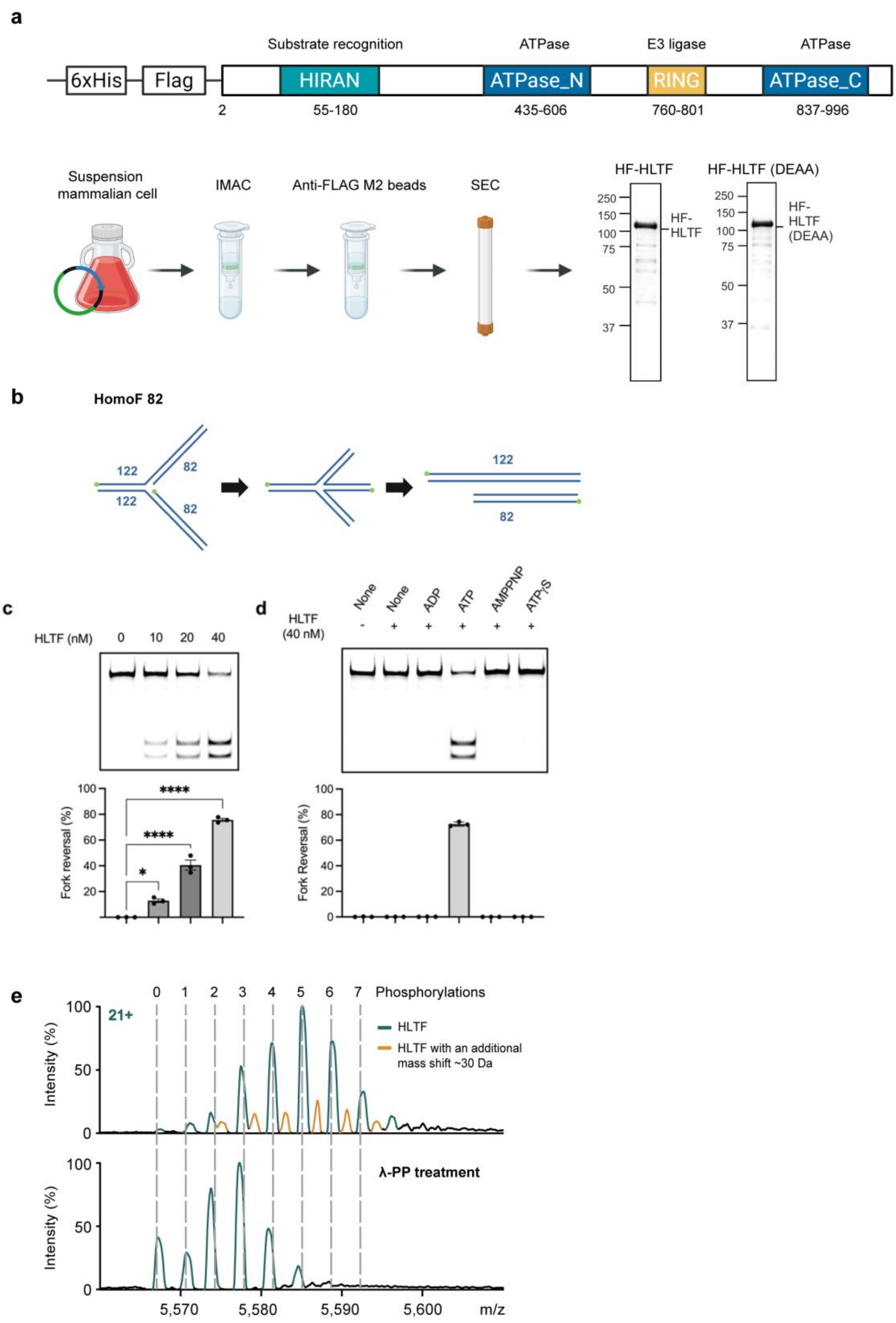

Figure S1. Characterization of purified recombinant HLTF. (**a**) Schematic overview of the construct design and purification workflow for human HLTF. Purified HLTF and HLTF (DEAA) analyzed in a 10% SDS-denaturing polyacrylamide gel with Coomassie Blue staining. (**b**) Schematic overview of fork reversal process from HomoF82 substrate. (**c**) Fork reversal assay with HomoF82 DNA titrating different concentration of HLTF. Data are shown as mean ± SEM from three replicates. One-way ANOVA, **P* < 0.05; *****P* < 0.0001. (**d**) Fork reversal assay with HomoF82 DNA supplemented with ATP or ATP

analogs. Data are shown as mean ± SEM from three replicates. **(e)** Mass spectrum of purified HLTF, with a magnified view of the 21^{+} charge state, showing the presence of a 30 Da and multiple phosphorylation mass shifts. IMAC, immobilized metal affinity chromatography; SEC, size-exclusion chromatography. Animated figure for (a) and (b) was created in BioRender. Chi, H. (2026) https://BioRender.com/vq6itcq.

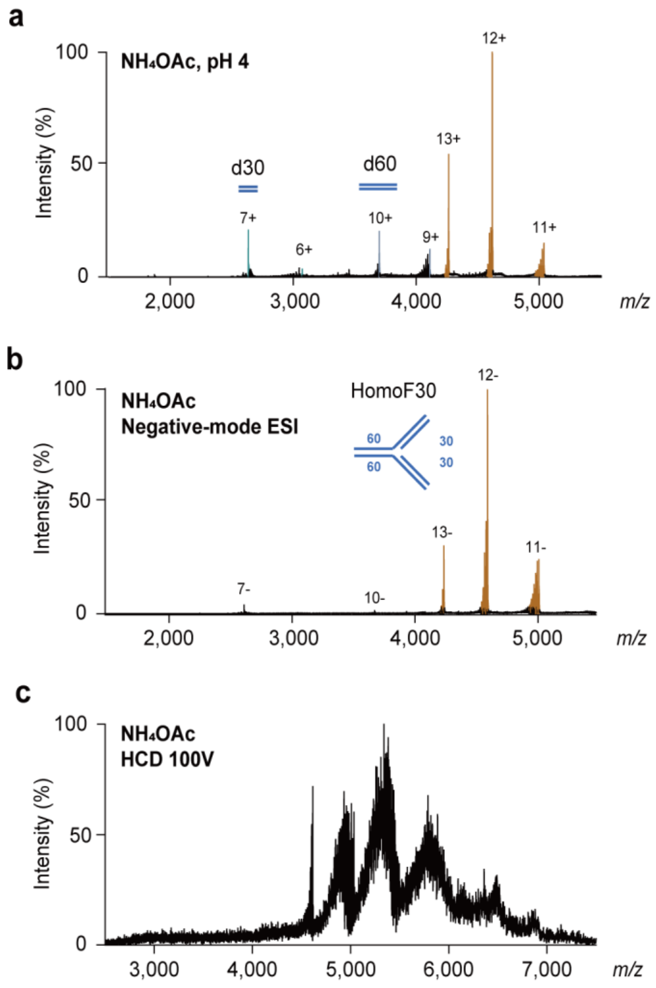

Figure S2. Characterization of DNA fork substrates. (**a**) Mass spectrum of HomoF30 acquired in ammonium acetate (350 mM) adjusted to pH 4.0. (**b**) Mass spectrum of HomoF30 acquired in ammonium acetate (350 mM, pH 7.0) under negative-mode ESI condition. (**c**) Mass spectrum of HomoF30 following collisional activation at 100 V in the HCD cell.
